# Effects of Exogenous Gibberellic Acid_3_ on Iron and Manganese Plaque Amounts and Iron and Manganese Uptake in Rice

**DOI:** 10.1371/journal.pone.0118177

**Published:** 2015-02-24

**Authors:** Yue Guo, Changhua Zhu, Lijun Gan, Denny Ng, Kai Xia

**Affiliations:** 1 College of Life Sciences, Nanjing Agricultural University, Nanjing, 210095, China; 2 CP Bio, Inc., 4802 Murrieta St., Chino, California, 91710, United States of America; Henan Agricultural Univerisity, CHINA

## Abstract

Gibberellins (GA) regulate various components of plant development. Iron and Mn plaque result from oxiding and hydroxiding Fe and Mn, respectively, on the roots of aquatic plant species such as rice (*Oryza sativa* L.). In this study, we found that exogenous gibberellic acid_3_ (GA_3_) spray decreased Fe plaque, but increased Mn plaque, with applications of Kimura B nutrient solution. Similar effects from GA_3_, leading to reduced Fe plaque and increased Mn plaque, were also found by scanning electron microscopy and energy dispersive X-ray spectrometric microanalysis. Reduced Fe plaque was observed after applying GA_3_ to the groups containing added Fe^2+^ (17 and 42 mg•L^-1^) and an increasing trend was detected in Mn plaques of the Mn^2+^ (34 and 84 mg•L^-1^) added treatments. In contrast, an inhibitor of GA_3_, uniconazole, reversed the effects of GA_3_. The uptake of Fe or Mn in rice plants was enhanced after GA_3_ application and Fe or Mn plaque production. Strong synergetic effects of GA_3_ application on Fe plaque production were detected. However, no synergetic effects on Mn plaque production were detected.

## Introduction

Mineral nutrients are chemical elements that plants obtain primarily from surrounding soil. They are needed for basic functions in plant metabolic, physiological, and developmental processes [1,2]. Plants depend on complex sensing and signaling mechanisms to detect external and internal concentrations of mineral nutrients [3]. Evolutionary changes in plants have included enhanced root growth [1,4,5], changes in expression and activity of ion transporters [6,7], and acidification of the surrounding soil to mobilize mineral nutrients [8]. Recent studies have identified plant hormones involved in regulation of mineral nutrient availability. Conversely, mineral nutrients influence hormone biosynthesis, suggesting a relationship between hormones and nutritional homeostasis. For instance, cytokinins and abscisic acid functioned in nitrate resupply experiments [3,9,10], ethylene acted in root hair regulation in response to low Fe supply [3], and auxin had bidirectional antagonistic effects with S deprivation signaling and upregulated K transporter accumulation [6,10–13]. GAs is essential plant hormones that affect nearly all aspects of higher plants growth and development [14,15]. There are also several investigations indicating that GAs is involved in the K [16, 17], P [4, 74] and Fe [1, 18, 75, 76] nutrition in plants.

Fe is an essential microelement for several plant processes, particularly chlorophyll biosynthesis. Fe mainly exists as insoluble ferric ions in oxygen-rich soil and approximately neutral pH conditions and is usually in insufficient quantities for plants [18]. Thus, plants have developed two main Fe-uptake mechanisms categorized as strategy I and strategy II [19]. In most monocotyledons (strategy II), phytosiderophores (mugineic acids) are secreted into the soil to chelate with ferric ions through TOM1 [20,21]. The Fe—mugineic acid complexes are absorbed by root cells through YELLOW-STRIPE1 [22,23]. In most dicotyledons (strategy I), Fe is acquired from the soil by *IRT1* (IRONREGULATED TRANSPORTER 1) and *FRO2* (FERRIC REDUCTION OXIDASE 2) localized in the root epidermis.

Iron and Mn are oxidized and their oxide/hydroxide products, known as Fe plaque and Mn plaque, respectively, are precipitated on the root surface of aquatic plant species such as rice, *Typha latifolia* L., and *Phragmites australis* Trin. These plaque result from oxidizing Fe^2+^ and Mn^2+^ to Fe^3+^ and Mn^3+^, respectively [24,25]. In root plaque, Fe is the primary element and Mn is a secondary element. Iron and Mn usually co-exist, since the redox potentials of precipitating Fe oxides and hydroxides are lower than those of Mn oxides [26–35]. Iron and Mn plaque have diverse environmental and ecological functions in adapting to flooding and other environmental stresses. Plaque can act as a barrier to oxygen loss, which, in turn, enhances oxygen supply to root meristems [36] and affects the number of rhizosphere microorganisms [37]. Iron and Mn plaque have been shown to increase the uptake of toxic and nutrient elements [38–40]. The overall effect of Fe plaque on plant uptake of nutrients or harmful ions may depend on the amount of Fe plaque on the plant root surfaces [32,40]. The aim of the present study was to (1) investigate the effect of exogenous gibberellic acid_3_ (GA_3_) on Fe and Mn plaque, and (2) examine the response of Fe and Mn uptake to GA_3_ application and Fe and Mn plaque treatments.

## Materials and Methods

### Plant materials and seedling growth

The japonica rice variety Nanjing 44 was used in this study. Seeds were detoxified in 2% NaClO for 5 min and cleaned in distilled water. They were then soaked for 1 d in distilled water, followed by germination on nets. After growth at 30°C for 5 d, uniform seedlings were selected and transplanted to 300-mL pots (15 seedlings per pot) containing Kimura B nutrient solution (KB, modified from Kimura B macronutrients and Arnon micronutrients). This nutrient solution contained the macronutrients (mM): (NH_4_)_2_SO_4_ (0.18), MgSO_4_·7H_2_O (0.27), KNO_3_ (0.09), CaNO_3_·4H_2_O (0.18) and KH_2_PO_4_ (0.09), and the micronutrients (μM): Na_2_EDTA-Fe(II) (20), MnCl_2_·4H_2_O (9), H_3_BO_3_ (46), Na_2_MoO_4_·4H_2_O (9), ZnSO_4_·7H_2_O (0.7) and CuSO_4_·5H_2_O (0.3). Concentrations of Fe^2+^ and Mn^2+^ were 1.2 and 0.5 mg·L^-1^, respectively. The pH of this solution was adjusted to 5.0 using 0.1 M HCl and 0.1 M KOH [41], and solution volume was restored daily and renewed every 3 d. The plants were cultured in a PGX-450C controlled environment growth chamber (Ningbo Sai Fu Instrument Co., Ltd., China) with a 14-h / 28°C day and 10-h / 22°C night regime, light intensity of 375 μm mol m^-2^s^-1^ and 65% relative humidity.

### Application of plant hormones and their inhibitors

Four-leaf seedlings were sprayed with 0.18 mM GA_3_, 0.12 mM uniconazole (S3307) and 100 mg·L^-1^ sodium bisulfate (NaHSO_3_) aqueous solutions, respectively. Control plants were treated with distilled water. Gibberellic acid_3_ was purchased from Jiangxi Xinrunfeng Biochemical Co., Ltd (Ji’an, China), S3307 was purchased from Sichuan Academy of Chemical Industry Research and Design (Chengdu, China) and NaHSO_3_ was purchased from Sinopharm Chemical Reagent Co., Ltd.

**The Fe and Mn plaque dose-response design after hormone treatments**. Four-leaf rice seedlings grown in KB (1.2 mg·L^-1^ Fe^2+^ and 0.5 mg·L^-1^ Mn^2+^, pH 5.0) were harvested at a GA_3_ concentration gradient (0.03, 0.06, 0.12, 0.18, and 0.24 mM) or its inhibitor, S3307 (0.04, 0.08, 0.12, 0.16, and 0.20 mM) 60 h after spraying. Treatments were compared with distilled-water-treated samples (0 mM).

**Fe and Mn plaque time-course design after hormone treatments**. Four-leaf rice seedlings grown in KB (1.2 mg·L^-1^ Fe^2+^ and 0.5 mg·L^-1^ Mn^2+^, pH 5.0) were treated with 0 or 0.18 mM GA_3_ foliar spray for 96 h. Plants not treated with GA_3_ were the control.

### Nutrient solution treatments

Four-leaf seedlings were grown in KB solution and, prior to Fe or Mn plaque induction, all seedlings were placed in deionized water for 12 h to minimize interference from other elements. They were then transferred into 300-mL KB solution with 0, 17, and 42 mg·L^-1^ of the Fe ion (Fe^2+^ as ferrous ammonium sulfate) or 0, 34, and 84 mg·L^-1^ of the Mn ion (Mn^2+^ as manganese sulfate monohydrate) for 60 h after GA_3_ or S3307 spray. The same nutrient solutions were supplemented every day for a constant culture solution volume. Solution pH was adjusted to 5.0 with 0.1 M HCl or 0.1 M NaOH to promote root growth. Control plants were treated with distilled water and KB solution. Ferrous ammonium sulfate [(NH_4_)_2_Fe (SO_4_)_2_·6H_2_O] and manganese sulfate monohydrate (MnSO_4_·H_2_O) were purchased from Shoude Apparatus Corporation (Nanjing, China).

### Extraction and determination of Mn and Fe on the root surface

Harvested plant material was divided into roots and shoots, and rinsed thoroughly in distilled water. Iron and Mn plaque deposited on the root surface were extracted using the dithionite-citrate-carbonate (DCB) method of Taylor and Crowder [42] and McLaughlin et al. [43]. The fresh roots were incubated for 3 h at 25°C in 45 mL of a solution (pH 6.5) containing 0.27 M sodium citrate (N_a3_C_6_H_5_O_7_·2H_2_O) and 0.11 M sodium bicarbonate (NaHCO_3_), with the addition of 3.0-g sodium dithionite (Na_2_S_2_O_4_). Na_2_S_2_O_4_ is a strong reducer in NaHCO_3_ solution and can reduce Fe^3+^ to Fe^2+^, while citrate in Na_3_C_6_H_5_O_7_·2H_2_O can form a complex with Mn^2+^ and Fe^2+^. These compounds remove Fe and Mn plaque from root surfaces. After filtration, the extraction solution was analyzed for Mn and Fe content using an inductively coupled plasma optical emission spectrometer (ICP-OES) (Optima 2100, Pekin Elmer, USA) after an appropriate dilution, and roots were oven dried at 80°C for 3 d and weighed before digestion.

### Scanning electron microscopy and energy dispersive X-ray spectrometric microanalysis

Roots pre-treated with GA_3_ or distilled water (control) were harvested after 60 h and quick-frozen and freeze-dried. After applying a gold coating, root segment images and element ratio measurements were obtained using a scanning electron microscope (SEM) (model S-3000N; Hitachi High-Technologies Corporation, Tokyo, Japan) equipped with an energy-dispersive X-ray detector (EDX) (Horiba Inc., Kyoto, Japan) as described by Chen et al. [41] and Xie et al. [44].

### GA_1/3_ isolation and analysis by enzyme-linked immunosorbent assay (ELISA)

Leaves of four-leaf rice seedlings were harvested at nine time points (0, 12, 24, 36, 48, 60, 72, and 96 h) after foliar application of exogenous GA_3_. The 0.5-g samples were weighed, frozen in liquid nitrogen, and maintained at—28°C before hormone extraction. GA_1/3_ extraction and purification prior to immunoassay were conducted according to previous reports [45–49]. The main steps were: extraction of homogenized samples at a rate of 5 ml g^-1^ fresh weight overnight at 4°C (with 10 mg· L^-1^ butylated hydroxytoluene to prevent oxidation) in 80% cold (v/v) aqueous methanol. The supernatants were collected after centrifugation at 10,000 × g (4°C) for 20 min. The crude extract was passed through a C_18_ Sep-Pak cartridge (Waters, Milford, MA, USA), and the filtrate was collected. A 400-μl aliquot of the filtrate was removed and dried under N_2_. The extraction residues were dissolved in 200-μl phosphate-buffered saline (PBS) (0.01 M, pH 9.2), adjusted to pH 8.5, and separated three times with an equal volume of ethyl acetate. The aqueous phase of the remaining extract was adjusted to pH 2.5 and extracted three times with equal volumes of ethyl acetate. The ethyl acetate extracts were collected and dried under N_2_. Then, the residue was re-dissolved in 200-μl PBS (0.01 M, pH 7.4) to analyze GA_1/3_. GA_1/3_ levels were determined by ELISA based on a monoclonal antibody (provided by Nanjing Agricultural University, Jiangsu, Nanjing, China), as described previously [48,49].

### Microscopy

Whole roots of four-leaf rice seedlings were excised and photographed with a digital camera (FinePix S7000, Fujifilm, Tokyo, Japan) 60 h after a foliar application of 0.18 mM GA_3_.

### Statistical analysis

Means and standard errors were computed for three independent experiments, with at least three replicates per experiment. Treatment differences were detected using *t*-tests or Duncan’s multiple range tests in the SPSS 19.0 statistical software package.

## Results

### GA_3_ affects Fe and Mn plaque contents in a dose/time-dependent manner

We examined GA_1/3_ content after a GA_3_ foliar spray over 96 h to investigate the effects of a 0.18 mM GA_3_ application on endogenous biologically active GA. GA_1/3_ content increased significantly during the initial 48 h, followed by a sharp decline at 60 h (S1 Fig.). Then, GA_1/3_ content decreased slightly to its lowest level of 741.56 pmol·g^-1^·FW at 96 h. Thus, the 0.18 mM GA_3_ application in our experimental system strongly stimulated production of endogenous GA_1/3_, which corresponded to previous studies [50, 51].

Fe and Mn plaques form a reddish-brown deposition of iron oxide/hydroxide and amorphous colloidal substances on the root surfaces of aquatic plants [25]. In our experiment, rice plants were treated with exogenous GA_3_ foliar spray for 60 h, and the color of the rice root surface changed to light reddish-brown compared with the white roots of control plants (S1 Fig.). The root plaque image was quite consistent with previous reports [25]. However, we did not detect a difference in root length between the GA_3_ and control lines.

**Fig 1:**
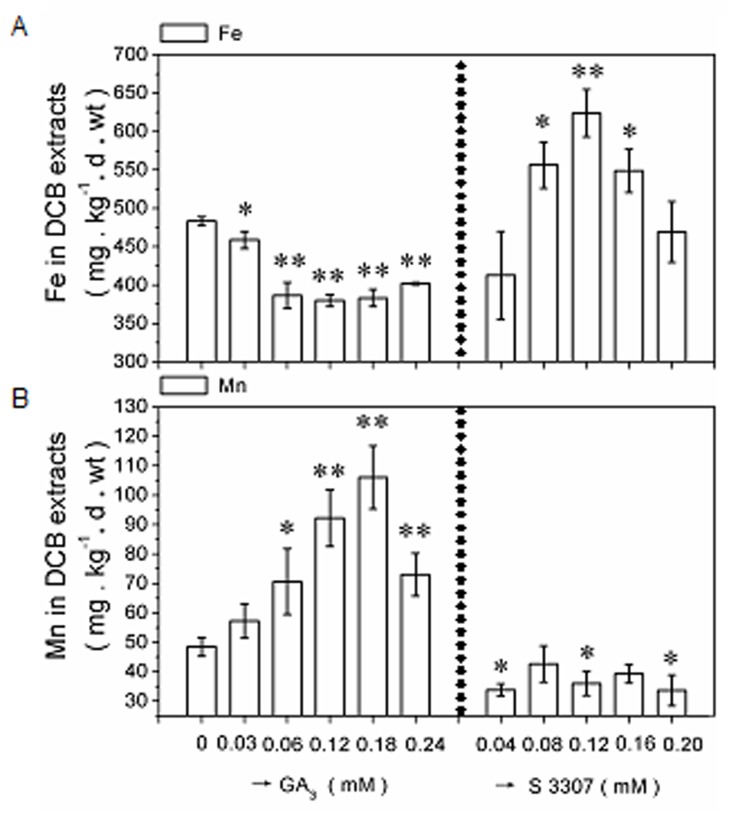
Dose response of Fe and Mn plaques to exogenous GA_3_ or S3307 treatment. Four-leaf rice seedlings grown in KB (1.2 mg·L^-1^ Fe^2+^ and 0.5 mg·L^-1^ Mn^2+^, pH 5.0) were harvested at a concentration gradient of GA_3_ (0.03, 0.06, 0.12, 0.18, and 0.24 mM) or its inhibitor, S3307 (0.04, 0.08, 0.12, 0.16, and 0.20 mM) spraying treatments after 60 h, and compared with distilled-water-treated samples (0 mM). The Fe (A) and Mn (B) plaques were extracted using the dithionite-citrate-carbonate (DCB) method and detected by ICP-OES. Data are the means ± SE of at least three independent experiments (n = 15) with similar results. * and ** significantly different between the distilled water (0 mM) and the exogenous GA_3_ or S3307 treatment at the *P* < 0.05 or 0.01 level according to a *t*-test.

Compared to the control, the application of exogenous 0.18mM GA_3_ resulted in a considerable decline (*P*<0.05 or *P*<0.01) in Fe plaque at 60 h after being spraying on KB-cultured plants (Fig. 1A). A GA biosynthesis inhibitor [52], S3307, was then applied to confirm the GA_3_ effects on Fe plaque production. Iron plaque content significantly increased after S3307 application at 0.08, 0.12, and 0.16 mM (Fig. 1A).

**Fig 2:**
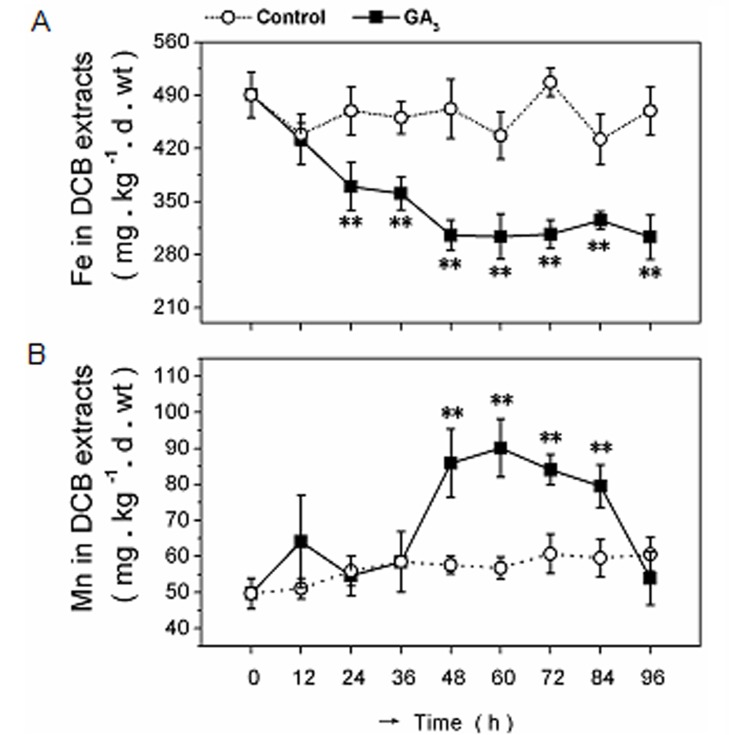
Time course of Fe and Mn plaques after exogenous GA_3_ treatment. Four-leaf rice seedlings grown in KB (1.2 mg·L^-1^ Fe^2+^ and 0.5 mg·L^-1^ Mn^2+^, pH 5.0) were treated with 0.18 mM GA_3_ foliar spray for 96 h. Plants without GA_3_ treatments were the control. The Fe (A) and Mn (B) plaques were extracted using the DCB method and detected by ICP-OES. Data are the means ± SE of at least three independent experiments (n = 15) with similar results. The control and exogenous GA_3_ treatments were compared using a *t*-test.

In the GA_3_ time-course experiment, the Fe content decreased after application of 0.18 mM GA_3_ during the initial 24 h of treatment, followed by a more-gradual decrease. The lowest Fe content was recorded at 48 h, when there was a plateau at 310 mg·kg^-1^·d·wt until 96 h (Fig. 2A). In the GA_3_ dose-response test, 0.06–0.24 mM GA_3_ significantly increased Mn plaque content, with a maximum response at 0.18 mM, compared to the control (Fig. 1B). Exogenous spraying of 0.04, 0.12, and 0.20 mM S3307 foliar significantly inhibited Mn plaque production (Fig. 1B). Regarding the time-course, Mn content increased slightly at 12 h, then increased markedly to a peak of 90 mg·kg^-1^·d·wt at 60 h (Fig. 2B). These results suggested that GA_3_ regulated Fe and Mn contents in a dose/time-dependent manner.

### GA_3_ regulates ions distribution of Fe and Mn plaque on rice root surfaces

**Fig 3:**
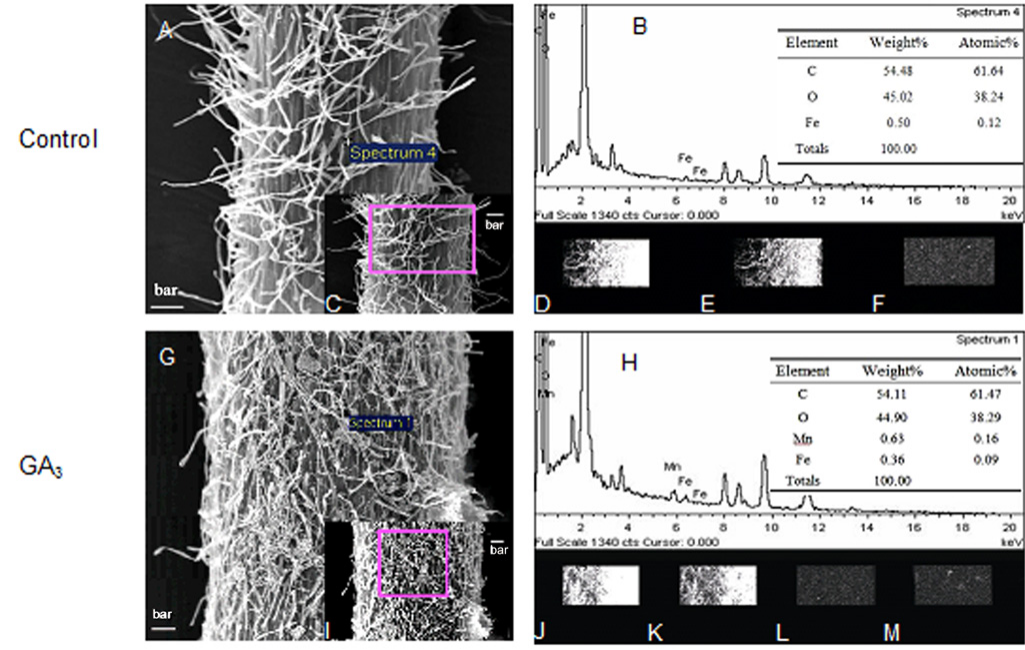
Scanning electron micrographs and energy-dispersive X-ray analysis of rice root surfaces after GA_3_ treatment. Four-leaf rice seedlings were grown in KB solution (1.2 mg·L^-1^ Fe^2+^ and 0.5 mg·L^-1^ Mn^2+^, pH 5.0) and harvested at the 60 h after spraying with 0.18 mM GA_3_ (G-M) or distilled water (A-F). Rice root morphology was assessed by scanning electron microscopy (SEM) with (G, H) or without (A, B) GA_3_ spraying. The images of any section on root surfaces marked in pink color after GA_3_ (I) or control (C) treatments and the energy-dispersive X-ray analysis (EDX) dissection of ion-distribution in pink region of root surfaces which was shown with white dots (D-F, J-M). These regions exhibited different nutrient compositions: C (D, J), O (E, K), Fe (F, L) and Mn (M). The micrographs are representative of the general morphology. Bar = 50 nm.

Exogenous application of 0.18 mM GA_3_ aqueous solution greatly increased Mn plaque content, which was deposited mainly on the root hairs (Fig. 3G), compared to the control (Fig. 3A). There was a small decrease in Fe content after GA_3_ treatment (accounted for 0.36% and 0.09% of weight and atomic, respectively) in comparison to the control (accounted for 0.5% and 0.12% of weight and atomic, respectively) according to spectrum analysis (Fig. 3H and B). The distribution of C, O, Fe and Mn on root surface sections of rice seedling plants at 60 h after various treatments was assessed using X-ray density maps (Fig. 3C–F and 3I–M). Compared to the control, more Mn (Fig. 3M) and less Fe (Fig. 3L) were present in the root sections of plants treated with GA_3_. Changes in Fe and Mn distribution are indicated by white dots, and compared with the brightness of C (Fig. 3D and J) and O (Fig. 3E and K). The absence of Mn in the control treatment was attributed to it being present at levels below the detection limits (Fig. 3H). These observations suggested that GA_3_ treatments decreased Fe plaque content, but increased Mn plaque content (Figs. 1 and 2).

### GA_3_ alters Fe and Mn plaque contents under additional Fe/Mn-inducing conditions

**Fig 4:**
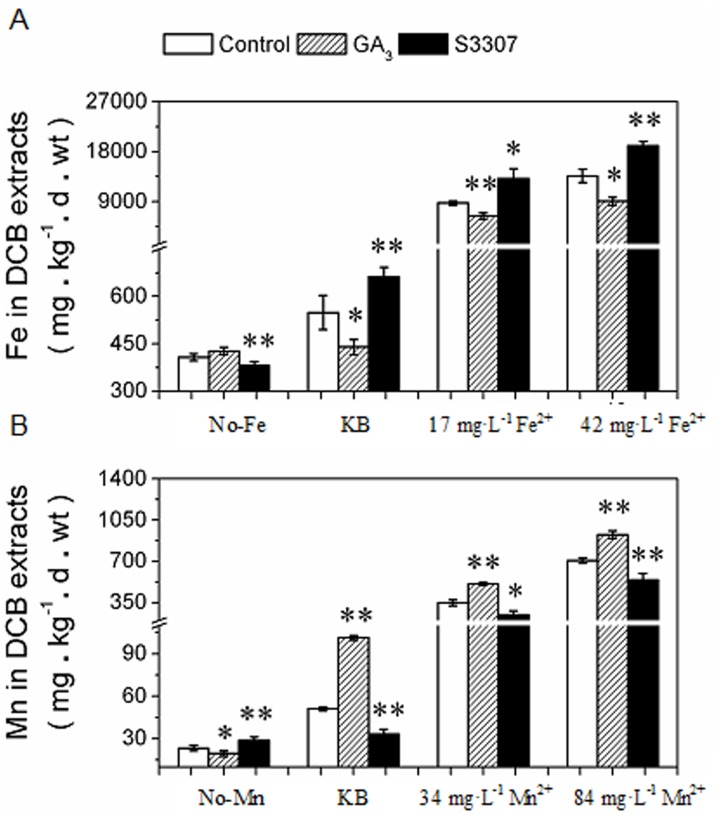
Effects of induction by application of exogenous plant hormones and metal ions on Fe and Mn plaque production. Four-leaf rice seedlings grown in KB (1.2 mg·L^-1^ Fe^2+^ and 0.5 mg·L^-1^ Mn^2+^, pH 5.0) and KB containing various concentrations of Fe^2+^ (zero Fe, 17 mg·L^-1^ and 42 mg·L^-1^, pH 5.0) or Mn^2+^ (zero Mn, 34 mg·L^-1^ and 84 mg·L^-1^, pH 5.0) were harvested at 60 h after spraying with 0.18 mM exogenous GA_3_ or 0.12 mM exogenous S3307. Plants that did not undergo GA_3_ or S3307 treatment served as the control. The Fe (A) and Mn (B) plaques were extracted using the DCB method and detected by ICP-OES. Data are the means ± SE of at least three independent experiments (n = 15) with similar results. The control and exogenous GA_3_ or S3307 treatments were compared using a *t*-test.

There was a dose-dependent increase in Fe plaque content in response to high Fe^2+^-inducing treatments of exogenous (NH_4_)_2_SO_4_·FeSO_4_·6H_2_O, compared to the KB and zero Fe treatments (Fig. 4). The same tendency was found for Mn^2+^-inducing treatments with exogenous MnSO_4_·H_2_O (Fig. 4), which is consistent with a previous report [38]. Compared to the control, GA_3_ treatments significantly decreased Fe plaque content at 17 and 42 mg·L^-1^ Fe ion (Fig. 4A).

The amount of Mn plaque following GA_3_ treatment increased in nutrient solutions containing concentrations (34 mg·L^-1^ and 84 mg·L^-1^) of the Mn ion, compared with the control (Fig. 4B). These results were similar to those regarding dose/time dependency (Figs. 1 and 2) and SEM-EDX tests (Fig. 3).

Application of 0.12 mM S3307 significantly increased the Fe content, but decreased the Mn content, with the exception of the zero Fe and zero Mn treatments, compared to the control (Fig. 4A-B). The effect of GA_3_ and S3307 on the zero Fe treatment was identical to that of nutrient solutions containing Mn, while the effect of the zero Mn treatment was similar to those of the Fe enrichment solution treatments (Fig. 4A-B). These results suggested that GA_3_ regulates the Fe and Mn plaque content in the presence of Supplementary Fe^2+^ and Mn^2+^, the efficiency of which was correlated closely with the Fe^2+^ and Mn^2+^ concentrations.

### GA_3_ or Fe plaque induction influences Fe uptake in rice seedlings

We further investigated the Fe content in rice seedlings following the GA_3_ and Fe plaque-inducing treatments. As reported previously, the Fe plaque induced by exogenous additional Fe^2+^ application appears to enhance Fe uptake in plants [29]. In our study, we found that solutions with additional Fe content increased Fe plaque production and significantly increased Fe content in the entire plant, compared to the KB treatment (Table 1). Following treatment with exogenous 0.18 mM GA_3_, shoot and total plant growth increased significantly for each nutrient treatment compared to the control, while the reverse was the case for root Fe uptake (Table 1). These results suggest that both GA_3_ and Fe plaque-inducing treatments promote Fe uptake by rice seedlings.

**Table 1:** Effects of exogenous GA_3_ and high-Fe plaque on Fe uptake by rice seedlings.

Treatments	Root (mg·kg^-1^·d·wt)		Shoot (mg·kg^-1^·d·wt)		Total plant (mg·kg^-1^·d·wt)	
	Control	GA_3_	Control	GA_3_	Control	GA_3_
KB	87±5^b^	76±4^c^	348±26^b^	492±36^b^	318±26^b^	420±28^b^
17 mg·L^-1^ Fe^2+^	200±12^a^	175±10^b^	1127±6^a^	1414±56^a^	977±42^a^	1216±39^a^
42 mg·L^-1^ Fe^2+^	216±7^a^	199±6^a^	1199±24^a^	1360±18^a^	1041±27^a^	1172±35^a^

GA_3_ was applied to rice seedlings in the presence of 17, 42, and 1.2 mg·L^-1^ Fe^2+^ in KB, and control plants were sprayed with distilled water. The rice roots were harvested at 60 h after treatment. Data are the means±SE from at least three independent experiments (n = 15) with similar results. Different letters within columns indicate significant differences (*P* < 0.05) among different cultivation solutions according to Duncan’s multiple-range test.

### GA_3_ or Mn plaque induction promotes Mn uptake in rice seedlings

Ye et al. [38] reported that in the presence of Fe or Mn, *Typha latifolia* adsorbed more Cu and had a higher proportion of Cu on its roots, especially roots harboring heavy Mn or Fe plaque. Manganese plaque had a greater Cu-adsorption capacity than Fe plaque, which suggests that Mn plaque enhances nutrient uptake. After detecting Mn plaque in rice roots, we found that the Mn treatments of exogenous addition increased Mn plaque content and significantly increased the Mn content in roots, shoots and the whole plants, compared to the KB treatment (Table 2). Exogenous GA_3_ increased the Mn content for all nutrient treatments compared to the control (Table 2). These results suggest that both GA_3_ and Mn plaque treatments increased Mn uptake by rice seedlings.

**Table 2:** Effects of exogenous GA_3_ and high Mn plaque content on Mn uptake by rice seedlings.

Treatments	Root (mg·kg^-1^·d·wt)		Shoot (mg·kg^-1^·d·wt)		Total plant (mg·kg^-1^·d·wt)	
	Control	GA_3_	Control	GA_3_	Control	GA_3_
KB	13.8±0.5^c^	15.6 ±0.3^c^	401±2^c^	496±14^c^	342±6^c^	393±13^c^
34 mg·L^-1^ Mn^2+^	22.8±1.8^b^	26.6 ±0.8^b^	1045±116^b^	1091±115^b^	807±81^b^	858±83^b^
84 mg·L^-1^ Mn^2+^	31.4±2.1^a^	35.1±1.8^a^	1426±20^a^	1477±8^a^	1132±15^a^	1172±36^a^

GA_3_ was applied to rice seedlings in the presence of 34, 84, and 0.5 mg·L^-1^ Mn^2+^ in KB, and control plants were sprayed with distilled water. The rice roots were harvested at 60 h after treatment. Data are the means±SE from at least three independent experiments (n = 15) with similar results. Different letters within columns indicate significant differences (*P* < 0.05) among different cultivation solutions according to Duncan’s multiple-range test.

### A glycolate oxidase inhibitor reduces GA_3_-induced Fe (Mn) plaque formation

**Fig 5:**
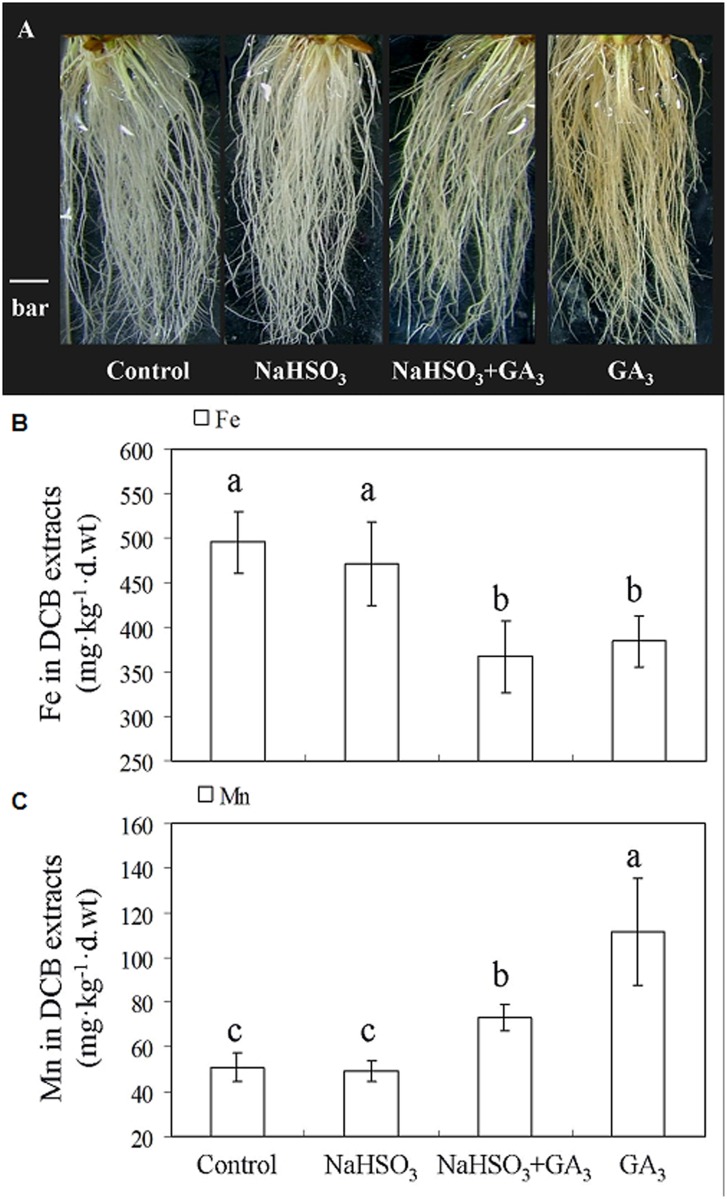
An inhibitor of glycolate oxidase influences the GA_3_-induced plaque changing. Four-leaf rice seedlings grown in KB (pH 5.0) were treated by foliar spray of exogenous GA_3_ (0.18mM), NaHSO_3_ (100mg·L^-1^) and GA_3_ with NaHSO_3_ (0.18mM, 100mg·L^-1^) respectively. Then roots were harvested to determine Fe and Mn plaque content after 60 h. The Fe (B) and Mn (C) plaques were extracted using the dithionite-citrate-carbonate (DCB) method and detected by ICP-OES. Data are the means ± SE of at least three independent experiments (n = 15) with similar results. Within each set of experiments, bars with different letters are significantly different *P*<0.05 according to Duncan’s multiple range tests. (A) Images were taken by digital camera after 60 h of the different treatments. Bar = 1cm.

The ability of the root to secrete O_2_ is related to the specific glycolic acid pathway in the rice root [53]. Iron plaque formation is due to the ability of the plant to release O_2_ into the rhizosphere [32]. We used NaHSO_3_ as an inhibitor of a key enzyme (glycolate oxidase) in the glycolic acid pathway, to investigate whether GA_3_-induced Fe (Mn) plaque formation was caused by the glycolic acid pathway. We pretreated four-leaf rice seedlings grown in KB (pH 5.0) with a foliar spray of exogenous GA_3_ (0.18 mM), NaHSO_3_ (100 mg·L^-1^), or GA_3_ with NaHSO_3_ (0.18 mM, 100 mg·L^-1^), respectively and compared the results with control lines treated with distilled water. We found that the NaHSO_3_ treatment alone failed to cause root color to turn light reddish-brown after 60 h. However, root color in the GA_3_ with NaHSO_3_ group was much less reddish-brown than that of the GA_3_ lines and more reddish-brown than the white roots of the control and NaHSO_3_ samples (Fig. 5A). Mn plaque content from the DCB extracts showed similar results; i.e., NaHSO_3_ inhibited Mn plaque formation, particularly under GA_3_-induced conditions (p < 0.05; Fig. 5C). However, the inhibition of Fe plaque content was not significant (Fig. 5B).

## Discussion

### GA_3_ changes ion composition in Fe and Mn plaque

Gibberellin was identified from *Gibberella fujikuroi*, a fungus that causes the ‘foolish-seedling’ disease of rice, which results in excessive elongation of infected plants [54]. Since that discovery, rapid progress has been made in our understanding of the biological effects of GA [55], but the relationship between GA and the Fe and Mn plaque is unknown.

Rice (*Oryza sativa* L.) oxidizes various compounds on its root surface and in the close vicinity of the roots, resulting in high tolerance for flooding or anoxic environments and root growth maintenance [56]. For example, rice roots oxidize the rhizosphere and detoxify phytotoxins, such as Fe^2+^ and Mn^2+^, by oxidizing root surfaces and the immediate rhizosphere [56–59]. The oxide/hydroxide products of Fe^2+^ and Mn^2+^ are known as Fe plaque and Mn plaque, respectively, and are deposited on the root surface of aquatic plant species. Iron plaque results from the plant releasing oxygen into the rhizosphere [32] and biological oxidation by microorganisms [60]. Mitsui [53] demonstrated a remarkable characteristic property called root oxygen (O_2_) secretion, which is likely linked with the specific glycolic acid pathway in rice roots. Thus, GA_3_ may affect Fe and Mn plaques through the oxidizing power of rice roots by influencing the glycolic acid pathway.

We found that exogenous GA_3_ treatment decreased the amount of Fe plaque, but increased that of Mn plaque in a dose- and time-dependent manner in KB-cultured plants (Figs. 1 and 2). The GA biosynthesis inhibitor S3307 increased Fe plaque content in 0.08, 0.12, and 0.16 mM KB treatments, but decreased Mn plaque in the 0.04, 0.12, and 0.20 mM treatments (Fig. 1).

The photographs show the color differences between the control with white roots and GA_3_ treated plants with light reddish-brown roots after 60 h (S2 Fig.), which is consistent with the widely accepted color characteristics of Fe and Mn plaques on roots [25]. The combination of 0.18 mM GA_3_, 0.12 mM S3307 and 60 h were used for the next experiment. Results from SEM-EDX (Fig. 3) were similar to the effects of GA_3_ on Fe and Mn plaque (Figs. 1 and 2); i.e., reduced Fe and increased Mn plaques after GA_3_ application (Fig. 3). GA_3_ is a long-distance signal transducer and one of the active GAs (GA_1_, GA_3_, GA_4_, and GA_7_) that stimulates increases in endogenous biologically active GA leading to leaf growth after exogenous application in rice [18, 50, 51]. We assayed endogenous GA_1/3_ in rice leaves over 96 h and found that GA_1/3_ content increased significantly from 12 to 60 h (S1 Fig.). Thus, the change in plaque content was due to exogenous GA.

Formation of Fe plaque on root surfaces is facilitated by the release of O and oxidants into the rhizosphere in aquatic plants. Under field conditions, plaque has been found on rice roots grown in flooded soil [25], *Juncus bulbosus* planted in acid lakes [61], and *Phragmites australis* collected from a field contaminated with coal mine drainage [62]. In the laboratory, however, plaque production can be induced by application of ferrous Fe (FeSO_4_) solution [29,30,63], nutrient solution containing Fe(OH)_3_ or FeSO_4_ [40,64], and (NH_4_)_2_Fe(SO_4_)_2_, FeSO_4_ and Fe(II)-EDTA [31,62,65]. To confirm the effects of GA_3_ on Fe and Mn plaque formation, we used complete KB containing 0.09 mM P, and added ferrous ammonium sulfate or manganese sulfate monohydrate to yield solutions of different Fe and Mn concentrations. The results supported the formation of increased Fe and Mn plaque amounts after additional application of exogenous Fe and Mn levels (Fig. 4). Application of GA_3_ caused decreases in Fe plaque content and increases in Mn plaque content (Figs. 1, 2 and 3), with the exception of the zero Fe and zero Mn treatments (Fig. 4). Exogenous S3307 spray treatment reversed the effects of GA_3_ on Fe and Mn plaques, with the exception of the zero Fe and zero Mn treatments (Fig. 4). With the exception of the zero Fe treatment, GA_3_ enhanced and S3307 reduced Fe plaque content. This was also the case for the Mn treatments followed by GA_3_ or S3307 application. Responses to zero Fe were similar to those to the Mn treatments, other than zero Mn (Fig. 4). Similarly, responses to solutions containing zero Mn were similar to those to the Fe treatments, with the exception of zero Fe (Fig. 4).

The above results were further confirmed, as exogenous GA_3_ induced Fe and Mn plaque formation on rice roots. Then, we attempted to identify the mechanism underlying this phenomenon. The glycolic acid pathway is a rice-specific metabolic process that releases O_2_ into the rhizosphere through the hydrolytic reaction of hydrogen peroxide [53]. NaHSO_3_ is a glycolate oxidase inhibitor analog (a key enzyme in the glycolic acid pathway) of hydroxypyridinemethanesulfonate [53,66–68]. We determined whether GA_3_-induced Fe (Mn) plaque formation was related to the glycolic acid pathway. As expected, applying NaHSO_3_ decreased Mn plaque contents in the DCB extract test and in the root system (Fig. 5A, C). However, no significant inhibition of Fe plaque content was observed, possibly due to absorption interference (Fig. 5B). Thus, we speculate that the effect of GA_3_ on Fe (Mn) plaque formation might be due to a variation in glycolic acid metabolism, particularly that of glycolate oxidase, which is a focus of our further studies.

### Mechanism for the mitigating effects of GA_3_ by uniconazole

The plant growth retardant uniconazole (S-3307, (E)-l-(4-CrJorophenyl)-4,4-dimethyl-2- (l,2,4-triazol-l-yl)-l-penten-3-ol) inhibits GA biosynthesis in rice plants with 50% growth retardation (S-3307 at 2.2 × 10^-7^ M) compared with that of the control [52]. This retardation was removed by applying GA (8.7 × 10^-5^ M). GA-like substances in rice shoots decreased following the S-3307 treatment, which was further confirmed by the main GAs (GA_1_ and GA_19_) in rice plants [52]. The inhibitory sites in GA biosynthesis and the comparative effects of its stereoisomers in a *Cucurbita maxima* cell-free system revealed that the transformation of [^14^C] mevalonic acid into GA_12_-aldehyde and GA_12_ was clearly inhibited, but kaurene accumulated after the S-3307 treatment [52, 69]. Further studies have demonstrated that kaurene, kaurenol, and kaurenal oxidation is inhibited by S-3307, which has no oxidation effect on kaurenoic acid. Then, the S-3307 reaction sites in GA biosynthesis were considered to be from kaurene to kaurenoic acid (three oxidation steps).

Thus, the mechanism for the mitigating effects of GA_3_ by S-3307 in our experiments was inhibition of endogenous GAs, mainly in the oxidation steps of GA biosynthesis from kaurene to kaurenoic acid [52, 69].

### Both GA_3_ and Fe and Mn plaque influence Fe/Mn uptake

GA is the major phytohormone promoting cell division and elongation as well as flowering and germination [70]. GA is a long-distance signal transducer. For example, GA produced in the upper shoots is involved in tissue reunion during wounding in cucumber hypocotyls [71]. It is also involved in xylem expansion in *Arabidopsis* hypocotyls [72]. Shoot-derived GA promotes *XSP30* expression (a xylem sap lectin) in cucumber roots [73]. In addition, numerous studies suggest that GA is involved in the mineral nutrient regulatory network, and mineral nutrient conditions also influence GA biosynthesis, suggesting a close association between hormonal stimuli and nutritional homeostasis [3]. Wakhloo [16] reported that *Solanum sisymbrifolium* buds released from inhibition in high-K plants only elongate following application of GA_3_. Chen et al. [17] reported that applying GA_3_ enhances K uptake in *Hong Mang Mai*. Higher sensitivity to GA at the first internode in the presence of K could increase elongation by increasing the amount of osmotic solute, which is important for *Hong Mang Mai* tolerance to deep-seeding conditions. GA contributes to Pi starvation responses, but does not regulate Pi starvation-induced changes in Pi uptake efficiency or the accumulation of Pi starvation-responsive gene transcripts via a DELLA-mediated signaling mechanism. Pi starvation reduces the bioactive GA level, and the accumulation of DELLA modulates the plant Pi-starvation response [4]. Ward et al. [74] found that P deficiency enhances accumulation of Fe, and that manipulating Fe supply improves the ability of Arabidopsis primary roots to tolerate P deficiency.

Several investigations have shown that GA is associated with Fe nutrition in plants. Kannan and Mathew [75] reported that GA_3_ promotes marrow bean roots to absorb and transport Fe to aboveground tissues. Marschner [1] reported that inhibiting primary roots is a well-documented response to an excess supply of some Fe-containing nutrients. Fe deficiency reduces GA content but does not affect Fe concentration in plant shoots [76]. Matsuoka [18] et al. demonstrated that GA regulates expression of Fe uptake-related genes by putative FIT-independent pathways under conditions of sufficient Fe and by FIT-dependent pathways under conditions of deficient Fe. For example, *IRT1*, *FRO2*, *bHLH038*, and *bHLH39* expression (*bHLH038* and *bHLH39* control expression of *IRT1* and *FRO2*) is promoted by GA_4_ in GA-deficient *Arabidopsis thaliana* mutants *(ga3ox1 ga3ox2*). Shoot-applied GA_4_ triggers induction of those genes, even in the *fit-2* mutant with reduced endogenous GA levels. Expression of Fe uptake-related genes, such as *bHLH038* and *bHLH39*, is lower in *ga3ox1 ga3ox2* compared with that in the wild-type under sufficient Fe conditions. However, the expression of all Fe uptake-related genes decreases under Fe-deficient conditions, and applying GA_4_ fails to induce FIT (encodes a transcription factor necessary for *IRT1* and *FRO2*) expression. Furthermore, PBZ decreases *IRT1* expression in the wild-type but not in the *fit-2* mutant.

Rice is a strategy II plant, but it possesses the OsIRT1 ferrous transporter, which absorbs Fe^2+^ [77] in addition to its uptake by the OsYSL15 transporter (strategy II—based Fe(III)-DMA) [77, 78, 79]. However, ferric-chelate reductase activity on the rice root surface is very low in contrast to that in strategy I plants [77, 79], indicating that it has acclimated to directly take up Fe^2+^, which is abundant in water-logged and anaerobic environments. Corresponding to these investigations on the association between GA and Fe, our data demonstrate that exogenous GA_3_ treatment increased Fe uptake in shoots and the entire plant, but decreased Fe content in roots. These results demonstrate the ability of GA_3_ to enhance uptake by a putative FIT-independent pathway under Fe-sufficient conditions and the possible translocation of Fe from roots to shoots in rice seedlings by GA functioning as a long-distance signal transducer (Table 1). Moreover, GA_3_ application increased Mn content throughout the plant (Table 2).

Fe and Mn plaques can act as a barrier to the uptake of toxic metals such as Cu, Ni, Mn, Pb, Cd and As [29,30,32,42,64,80]. In contrast, some studies on the growth of rice indicated that roots with Fe plaque adsorbed more Fe, but less P, than roots without plaque and in the presence of excess Zn [63]. Ye et al. [38] reported that Fe plaque increased Cu uptake in roots, but it had no influence on Cu translocation from roots to shoots in *Typha latifolia* plants. Iron plaque can act as a reservoir for nutrients such as Ca and P if they are deficient [4]. It has been suggested that the effects of Fe plaque on nutrient uptake or contaminants depend on the amount of Fe plaque on the root surfaces [32,39] and the ionic species involved [30].

In the present study, the presence of large amounts of Fe plaque, induced by additional application of exogenous Fe levels, significantly increased the Fe contents of roots, shoots and the whole plants, compared to the KB treatment (Table 1). Manganese plaque induced by additional application of exogenous level of Mn greatly enhanced Mn uptake compared with the KB treatment (Table 2). Moreover, we found that GA_3_ and Fe plaque increased the Fe uptake efficiency. No such effect was evident for the combination of GA_3_ and Mn plaque (Table 1 and Table 2). Since previous studies indicated that nutrients uptake was related to respiration in rice plants [53]. Although the mechanism behind this phenomenon has not been elucidated, we believe that the putative FIT-independent pathways and the energy alteration of root system under GA induction are needed further investigation.

Iron precipitated on root surfaces is composed mainly of goethite for *Juncus bulbosus* [61] or ferrihydrite (approx. 63%) for *Phalaris arundinacea* [37], which depends on the species and growth conditions in wetland fields. Under laboratory conditions, Fe plaque was present as an amorphous coating on roots with an uneven distribution [62,65], compared with the continuous precipitation in the field [37]. These studies revealed that Fe and Mn plaque are produced by a chemical deposition process; therefore, exogenous GA_3_ treatment should have identical effects on Fe plaque and Mn plaque in rice seedlings grown in KB (Figs. 1–4). However, our results showed that the amount of Mn plaque increased, but that of Fe plaque declined (Fig. 4). The increase in Fe plaque might be overshadowed by the considerable Fe uptake and translocation to shoots (Table 1).

Overall, the evidence provided here further confirmed that GA_3_ application decreased the Fe, but increased the Mn content in Fe and Mn plaques. Fe uptake was enhanced by both GA_3_ application and the presence of Fe plaque, and Mn uptake was enhanced by both GA_3_ application and the presence of Mn plaque on rice seedlings. Fe and Mn uptake might be related to the effects of plaque on the GA_3_ treatments. It is also possible that GA_3_ directly promotes Fe and Mn uptake through putative FIT-independent pathways or energy alteration in root system. The results presented in this report are important for both fundamental and applied plant biology. The synergistic effect between GA_3_ and Fe plaque is important (Table 1), and could contribute to the development and popularity of hormone and Fe fertilizer compounds for crop production.

## Supporting Information

S1 Fig Time course of GA_1/3_ content after exogenous GA_3_ treatment in rice leaves. Four-leaf rice seedlings grown in KB were treated with 0.18 mM GA_3_ foliar spray for 96 h. Plants not treated with GA_3_ were the control. GA_1/3_ was extracted and determined by ELISA. Data are means ± SE of at least three independent experiments (n = 15) with similar results. The control and exogenous GA_3_ treatments were compared using the *t*-test.
(TIF)

S2 Fig Morphology of iron and manganese plaques on root surfaces after exogenous GA_3_ induced treatment. Four-leaf rice seedlings grown in KB (pH 5.0) were pre-treated by spraying exogenous GA_3_ (0.18 mM) and compared with control samples (distilled water treatment). A photograph was taken after 60 h of GA_3_ or distilled water treatment. Bar = 0.5 cm.
(TIF)
