# Supplementary figures and images for: Effects of Exogenous Gibberellic Acid3 on Iron and Manganese Plaque Amounts and Iron and Manganese Uptake in Rice
Source: PLoS One. 2015 Feb 24;10(2):e0118177. doi: 10.1371/journal.pone.0118177 (PMC4339979; doi:10.1371/journal.pone.0118177)

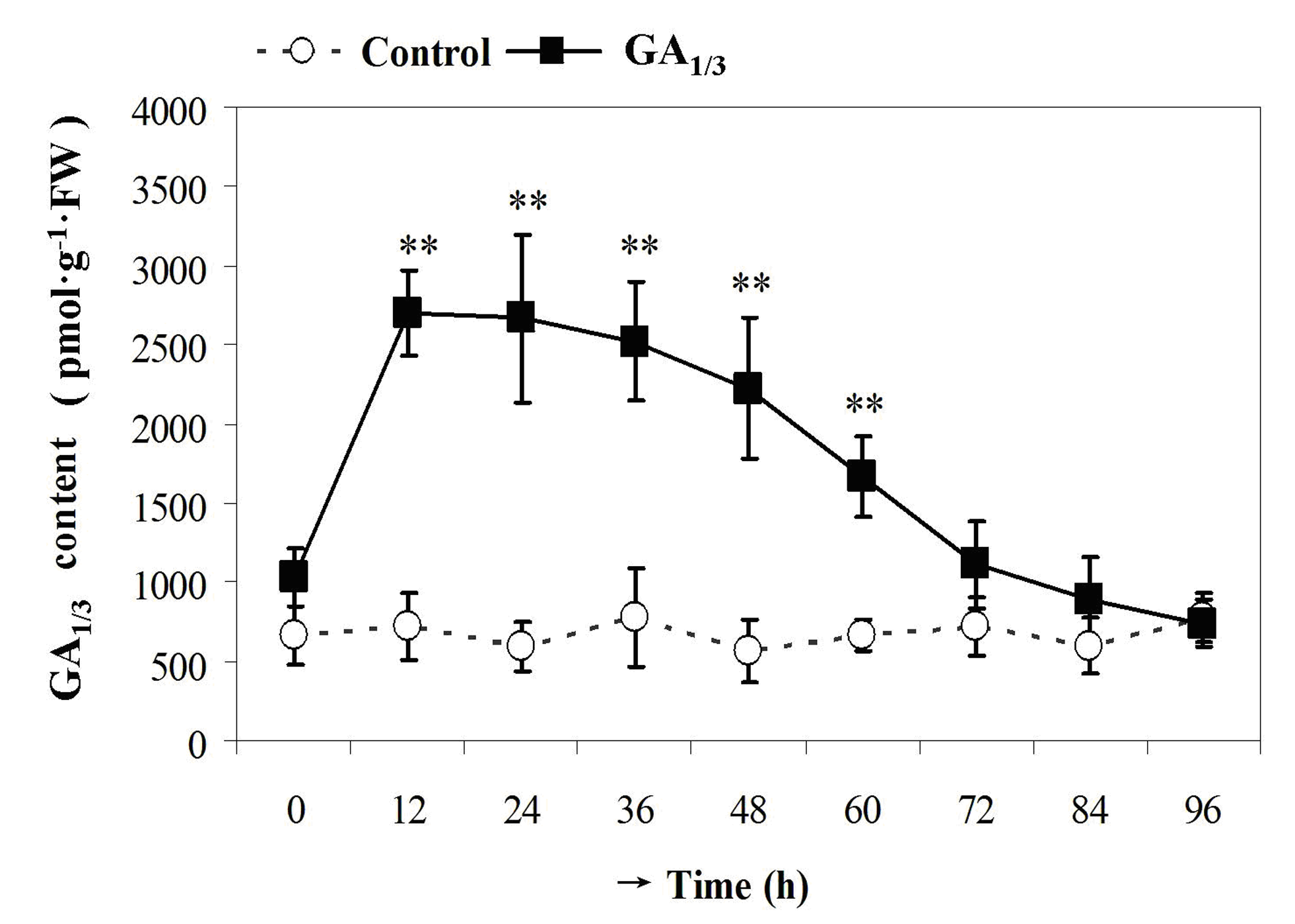

Supplement: S1 Fig — Four-leaf rice seedlings grown in KB were treated with 0.18 mM GA3 foliar spray for 96 h. Plants not treated with GA3 were the control. GA1/3 was extracted and determined by ELISA. Data are means ± SE of at least three independent experiments (n = 15) with similar results. The control and exogenous GA3 treatments were compared using the t-test. (TIF) [file pone.0118177.s001.tif]

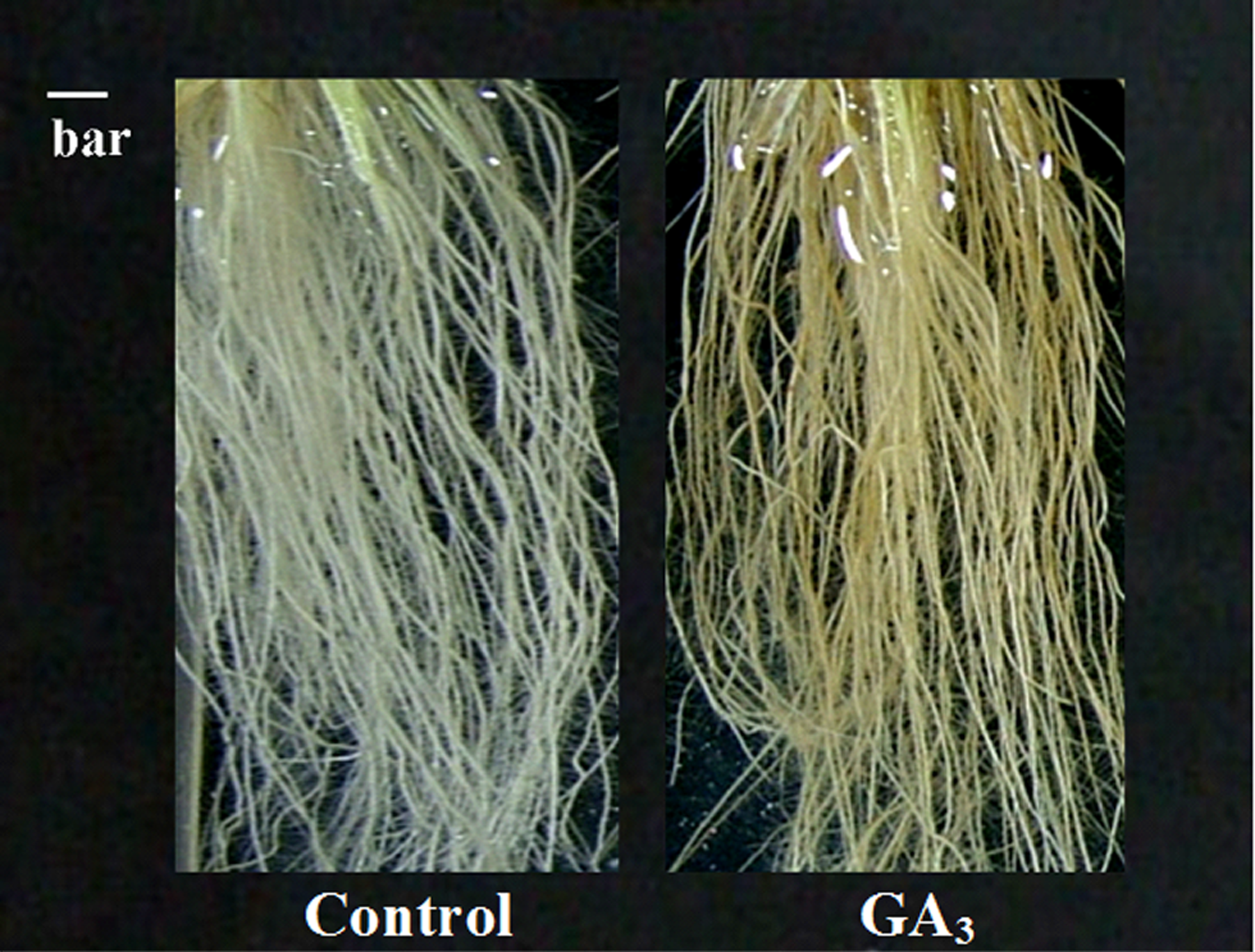

Supplement: S2 Fig — Four-leaf rice seedlings grown in KB (pH 5.0) were pre-treated by spraying exogenous GA3 (0.18 mM) and compared with control samples (distilled water treatment). A photograph was taken after 60 h of GA3 or distilled water treatment. Bar = 0.5 cm. (TIF) [file pone.0118177.s002.tif]
